# Editorial: C-Reactive Protein in Age-Related Disorders

**DOI:** 10.3389/fimmu.2018.02745

**Published:** 2018-11-21

**Authors:** Mark Slevin, Blanca Molins

**Affiliations:** ^1^Faculty of Science and Engineering, School of Healthcare Science, Manchester Metropolitan University, Manchester, United Kingdom; ^2^Institute of Dementia and Neurological Aging, Weifang Medical University, Weifang, China; ^3^University of Medicine and Pharmacy, Târgu Mureş, Romania; ^4^Institut d'Investigacions Biomèdiques Agustí Pi i Sunyer, Hospital Clínic de Barcelona, Barcelona, Spain

**Keywords:** dissociation, c-reactive protein, cell signaling, inflammation, biomarker

Over the last decade, native pentameric C-reactive protein (pCRP), and its biologically active dissociated monomer monomeric CRP (mCRP), have hit the headlines as it has become realized that they may not play such a passive role in disease development and pathophysiology as was once thought. In this series we have composed the thoughts and research of the most eminent scientists in the field of CRP-research in a mixture of original articles and reviews on the subject with a view to creation of an archive of current knowledge and understanding.

Both acute and chronic inflammation are hallmarks of potential injury and illness and are critical pathophysiological fingerprints of major disease development such as in cardiovascular disease, diabetes, obesity, and cancer (Gupta et al.). Circulating levels of pCRP have been used in conjunction with other biomarkers to measure accurately systemic levels of inflammation but too often only poorly, the prediction of future pathobiological events or consequences of the acute immune response. As our understanding of the biological importance of mCRP has increased, so has the suggestion that measurement of its circulating levels associated with microparticles, macrophages, and platelets of plasma, in patients with both acute and chronic inflammatory conditions could represent a much more accurate event predictor. Here, for the first time, Zhang et al. have produced a quantifiable ELISA assay accurately measuring mCRP levels in plasma. Furthermore, they went on to test this on a small cohort of patients with skin-related immune disorders showing that mCRP indeed predicted the course of illness far better than pCRP measurements. In acute coronary syndromes, autoimmune diseases (e.g., Lupus, giant cell arteritis) and possibly even some solid tumors, measurements of plasma mCRP may prove to be a significantly better prognostic indicator (1, 2).

Recent publications have highlighted important novel biological mechanisms associated with both native pCRP and mCRP signaling and pathophysiological action. For example, the work of Wu et al. (3) and Thiele et al. on understanding CRP conformational changes associated with induction of inflammation, and lipid binding details to apoptotic cell membranes by Alnaas et al. (4). In this series, we have included a group of focussed reviews relating the current state of knowledge of the role of CRP in major disease progression. Articles by Molins et al. Chirco and Potempa and Fernandez-Robredo et al. have discussed local inflammation and immune activation in age-related macular degeneration (AMD), a major cause of blindness, highlighting its pathogenic role via activation of complement; and identifying an association of A69S SNIP with high levels of CRP and degeneration in wet AMD.

Other reviews have discussed the impact of cellular and tissue deposition of mCRP on atherosclerotic plaque development, dementia and AMD (McFadyen et al.), and others the inflammatory links to diabetes and Parkinson's (Luan and Yao). Previously cited work by Strang et al. (5) and Slevin et al. (6) have already provided strong evidence of a possible causative involvement of mCRP in development of beta-amyloid plaques and neurodegeneration-associated cognitive impairment linked to Alzheimer's and dementia, suggesting that CRP manipulation could represent a future therapeutic target for helping to protect against development of these conditions.

Sproston and Ashworth discussed details of a novel mechanism for a role of CRP in response to infection, recruitment of leukocytes (also shown by Thiele et al. 7 in an ischemia-reperfusion injury model) and induction of nitric oxide (NO) (mCRP), following this up with a detailed original article measuring CRP associated changes in NO and iNOS using a monocyte-macrophage model of aging and inflammation. Additional reviews in our series have focussed on the ability of mCRP to activate endothelial cells, stimulating angiogenesis, increase plaque instability in coronary artery disease and potentially catalyze thrombosis leading to myocardial infarction (Badimon et al.). Di Napoli et al.. also discussed the utility of CRP as a biomarker for, and predictor of both long and short-term outcomes in intracerebral hemorrhage. Release of CRP and possible accumulation of mCRP in the brain after stroke, could contribute significantly to inflammation during the acute phase, and neurodegeneration thereafter (8) and hence further investigation of its role in these processes and the others described above is warranted.

Finally, we include in this series a group of articles focussed on detailing distinct novel biological properties of CRP. Sudhakar et al. showed increased CRP in non-morbidly obese individuals and preferred mCRP binding to the leptin receptor in human plasma and this could link to low-grade systemic infection on over-weight individuals. Jia et al. demonstrated binding of mCRP to receptor activator of NF-κB associated with blocking osteoclast differentiation with potential protective action in joint inflammation associated with rheumatoid arthritis. On the other hand Jimenez et al. showed that CRP may also play a role in the maintenance of peripheral T cell tolerance as they showed the ability of CRP to suppress development, maturation, and function of dendritic cells. Interest has arisen with regard to the potential interaction between CRP and nicotinic/acetylcholine receptors. CRP is known to affect innate immunity operating through the nicotinic acetylcholine receptors and here, Richter et al. (9) showed that pCRP associated with phosphocholine, could block macrophage-induced cytokine release potentially protecting trauma-associated injuries *in vivo*. In contrast, mCRP potently stimulates macrophage-associated inflammation, and further work detailed in this series showed that acetylcholine inclusion, within an *in vitro* model of inflammation, could effectively block mCRP-induced production of tumor necrosis factor-alpha (10).

A detailed analysis of crystal binding structure of mCRP by Caprio et al. concluded that various phospholipase A2 inhibitors might have therapeutic potential to block the early interaction of the molecule at the cell membrane thus negating activation of harmful biological effects. Future work could focus on effective prevention of mCRP binding to activated cell surfaces; or even more excitingly, either systemically or in a targeted form, blocking the primary pCRP-mCRP dissociation especially during acute inflammatory conditions.

In conclusion, the series provides an encyclopedia of current knowledge within the field of CRP research, it highlights new findings and major links to critical disease processes. It defines clear and opposing actions of the pentameric protein when compared with the dissociated monomer, and it expresses the complexity of cell and tissue specific interactions of this highly conserved molecule. Publications within this topic will undoubtedly increase over the coming years and we hope this collection will serve as a valuable reference for this future investigation.

## Author contributions

All authors listed have made a substantial, direct and intellectual contribution to the work, and approved it for publication.

### Conflict of interest statement

The authors declare that the research was conducted in the absence of any commercial or financial relationships that could be construed as a potential conflict of interest.
